# Perceptions of Artificial Intelligence in Medicine Among Newly Graduated Interns: A Cross-Sectional Study

**DOI:** 10.7759/cureus.71216

**Published:** 2024-10-10

**Authors:** Ali H Sanad, Aalaa S Alsaegh, Hasan M Abdulla, Abdulla J Mohamed, Ahmed Alqassab, Sayed Mohamed A Sharaf, Mohamed H Abdulla, Sawsan A Khadem

**Affiliations:** 1 Faculty of Medicine, Ain Shams University, Cairo, EGY; 2 Faculty of Medicine, Mansoura University, Mansoura, EGY; 3 Faculty of Medicine, First Moscow State Medical University, Moscow, RUS; 4 School of Medicine, Zhejiang University, Hangzhou, CHN; 5 Department of Radiology, Salmaniya Medical Complex, Manama, BHR

**Keywords:** ai in medicine, artificial intelligence, data privacy, healthcare, job displacement, medical education, medical interns, patient care, perceptions

## Abstract

Background

Artificial intelligence (AI) is rapidly transforming the healthcare sector, enhancing clinical decision-making, improving patient outcomes, and streamlining operations. Despite its promise, the integration of AI raises important questions about ethical considerations, data privacy, and implications for healthcare professionals.

Methods

This cross-sectional study utilized an online survey to assess the perceptions of newly graduated interns applying to post-graduate programs under the Saudi Commission for Health Specialties. A total of 349 participants were recruited through social media and professional networks. The structured questionnaire included sections on demographic information, awareness of AI, perceived impacts, concerns, training experiences, and future perspectives. Data were analyzed using descriptive and inferential statistics.

Results

The participants (N=349) were predominantly aged 20-25 years (142, 40.7%) with a higher representation of females (215, 61.6%). Awareness levels varied, with 65 participants (18.6%) reporting not being familiar with AI while 146 participants (41.8%) identified as familiar. A majority perceived AI positively, believing it improves patient diagnosis (114, 32.7%) and reduces medical errors (129, 36.9%). However, significant concerns emerged regarding data privacy (140, 40.1%) and job displacement (110, 31.5%). Notably, 189 participants (54.2%) reported no formal training in AI, highlighting a gap in preparedness.

Conclusions

The study reveals a mix of optimism and concern among newly graduated interns regarding AI in medicine. There is a critical need for enhanced training and education on AI technologies within medical curricula to prepare future healthcare professionals adequately. Addressing the opportunities and challenges posed by AI can foster a collaborative healthcare environment that prioritizes patient care while maintaining the human element of practice.

## Introduction

Artificial intelligence (AI) is rapidly transforming various sectors, with the healthcare industry being at the forefront of this technological revolution. AI encompasses a range of technologies, including machine learning, natural language processing, and data analytics, which have the potential to enhance clinical decision-making, improve patient outcomes, and streamline healthcare delivery [1-3]. As healthcare systems worldwide face increasing demands for efficiency and quality, the integration of AI solutions has become a focal point for innovation and improvement.

In recent years, AI applications have demonstrated significant promise in diverse areas such as diagnostic imaging, predictive analytics, personalized medicine, and administrative workflows [4,5]. For instance, AI algorithms can analyze medical images with remarkable accuracy, assisting radiologists in detecting anomalies that may be missed by the human eye [4-7]. Moreover, predictive models powered by AI can analyze patient data to identify individuals at risk for conditions such as diabetes or cardiovascular diseases, enabling proactive interventions [6,7].

Despite the evident advantages, the integration of AI into medical practice raises important questions regarding ethical considerations, data privacy, and the implications for healthcare professionals [5-8]. Concerns about job displacement, biases in AI algorithms, and the potential erosion of the doctor-patient relationship are prevalent among stakeholders. Additionally, the readiness of medical interns and newly graduated physicians to embrace and utilize AI technologies effectively remains a critical area of inquiry [6,9].

As newly graduated interns are poised to enter a workforce increasingly influenced by AI, understanding their perceptions of this technology is vital. Their views may significantly shape the future integration of AI in clinical settings, as they represent the next generation of healthcare providers who will be tasked with leveraging these advancements in their practice. This study aims to explore the perceptions of newly graduated interns in Bahrain regarding the use of AI in medicine, focusing on their awareness, perceived impacts, concerns, and preparedness to engage with AI technologies. By gaining insights into these perspectives, the findings will contribute to the ongoing dialogue about AI's role in healthcare and inform strategies for training and policy development.

## Materials and methods

Study design

This study employed a cross-sectional design utilizing an online survey to assess the perceptions of newly graduated interns regarding the use of AI in medicine. The survey was designed to capture demographic information, awareness levels, perceived impacts, concerns, training experiences, and future perspectives related to AI in healthcare. The Salmaniya Medical Complex Ethics Committee issued approval 108-031024.

Study participants

The target population for this study comprised newly graduated interns who are in the process of applying to post-graduate programs under the Saudi Commission for Health Specialties. The selection criteria included interns who had graduated within the last two years and were actively seeking further medical training. The participants were recruited through various channels, including social media platforms, medical schools, and professional networks. The calculated sample size for this study was 250 participants, determined based on statistical power calculations. This sample size was deemed adequate to allow for subgroup analyses and to enhance the generalizability of the findings.

Survey instrument

A structured questionnaire was developed specifically for this study, incorporating both closed and open-ended questions. The questionnaire was divided into several sections: demographic information, awareness of AI, perceived impacts of AI on patient care, concerns about AI, training received, and future perspectives on AI in healthcare. The questionnaire was pretested for clarity and comprehensiveness, and necessary adjustments were made based on feedback from a pilot group of interns.

Data collection

Data were collected using an online survey platform, ensuring accessibility and convenience for the participants. The survey link was disseminated via email and social media, along with an explanatory statement detailing the purpose of the study, voluntary participation, and confidentiality assurances. The participants were informed that the completion of the survey implied consent to participate. The survey remained open for a duration of four weeks, during which reminders were sent to encourage participation. Upon the completion of data collection, responses were exported for analysis using statistical software. Descriptive statistics were computed for demographic variables and survey responses, with frequencies and percentages reported.

## Results

Demographic information of the participants

The study included a total of 349 participants. The demographic characteristics are summarized in Table 1. The majority of the participants were aged 20-25 years (142, 40.7%), followed by those aged 26-30 years (98, 28.1%) and 31 years and above (109, 31.2%). In terms of gender, 134 participants (38.4%) were male, and 215 (61.6%) were female. Most participants graduated in 2023 (204, 58.4%).

**Table 1:** Demographic information of the participants (N=349) Data are represented as n (%), where n indicates the number of participants and % represents the percentage of the total sample (N=349)

Demographic variable		Frequency (N)	Percentage (%)
Age	20-25 years	142	40.7%
	26-30 years	98	28.1%
	31 years and above	109	31.2%
Gender	Male	134	38.4%
	Female	215	61.6%
Year of graduation	2022	145	41.6%
	2023	204	58.4%

Awareness of AI in medicine

The participants' awareness of artificial intelligence in the medical field is summarized in Table 2. Of the respondents, 65 participants (18.6%) reported being not familiar with AI, indicating that a significant portion of interns may lack exposure to this technology. Conversely, 88 participants (25.3%) stated that they were somewhat familiar with AI, while a majority of 146 participants (41.8%) identified as familiar with AI applications in healthcare. Notably, 50 participants (14.3%) claimed to be very familiar with AI, suggesting that there is a range of awareness levels among the interns. This variance underscores the need for enhanced educational initiatives regarding AI in medical training.

**Table 2:** Awareness of AI in medicine (N=349) Data are represented as n (%), where n indicates the number of participants and % represents the percentage of the total sample (N=349) AI: artificial intelligence

Awareness Level	Frequency (N)	Percentage (%)
Not familiar	65	18.6%
Somewhat familiar	88	25.3%
Familiar	146	41.8%
Very familiar	50	14.3%

Perceived impact of AI on patient care

The perceptions of the participants regarding the impact of AI on patient care are detailed in Table 3. A considerable number of interns believed that AI improves patient diagnosis, with 114 participants (32.7%) agreeing strongly with this statement. Additionally, 129 participants (36.9%) agreed that AI assists in reducing medical errors, highlighting a general optimism about the benefits of AI in clinical settings. However, opinions varied, as indicated by the 67 participants (19.2%) who remained neutral on this issue. Furthermore, 124 participants (35.5%) felt that AI can improve patient outcomes, illustrating a recognition of AI's potential to enhance healthcare quality.

**Table 3:** Perceived impact of AI on patient care (N=349) Data are represented as n (%), where n indicates the number of participants and % represents the percentage of the total sample (N=349) AI: artificial intelligence

Statement	Strongly disagree (n, %)	Disagree (n, %)	Neutral (n, %)	Agree (n, %)	Strongly agree (n, %)
AI improves patient diagnosis	30 (8.6%)	45 (12.9%)	60 (17.2%)	114 (32.7%)	100 (28.7%)
AI assists in reducing medical errors	20 (5.7%)	40 (11.5%)	60 (17.2%)	129 (36.9%)	100 (28.7%)
AI can improve patient outcomes	25 (7.2%)	30 (8.6%)	50 (14.3%)	124 (35.5%)	120 (34.4%)

Concerns about AI in medicine

Concerns regarding the integration of AI into healthcare practices are presented in Table 4. The predominant concern among the participants was related to data privacy and security, with 140 participants (40.1%) expressing apprehension about potential breaches and the misuse of sensitive patient information. Job displacement was another significant concern, noted by 110 participants (31.5%), indicating fears about the future roles of healthcare professionals in an increasingly automated environment. Ethical implications surrounding AI applications were also highlighted by 90 participants (25.8%), underscoring the need for careful consideration of AI's role in clinical decision-making.

**Table 4:** Concerns about AI in medicine (N=349) Data are represented as n (%), where n indicates the number of participants and % represents the percentage of the total sample (N=349) AI: artificial intelligence

Concern	Frequency (N)	Percentage (%)
Job displacement	110	31.5%
Ethical implications	90	25.8%
Overreliance on technology	55	15.7%
Data privacy and security	140	40.1%
Disparities in access	78	22.4%

Training and preparedness regarding AI

A total of 160 participants (45.8%) reported having received some form of training related to AI applications, while 189 participants (54.2%) had not received any training. This lack of training raises questions about the preparedness of newly graduated interns to effectively integrate AI into their practice. When asked about their preparedness, 160 participants (45.8%) felt adequately prepared to work with AI technologies, yet 45 participants (12.9%) reported feeling not prepared at all, indicating a gap that may need to be addressed through targeted educational efforts.

Future perspectives on AI in healthcare

The future perspectives of the participants regarding AI in healthcare are illustrated in Table 5. A majority of 129 participants (36.9%) strongly agreed that AI will significantly change healthcare roles, suggesting a recognition of the transformative potential of AI. However, 99 participants (28.4%) expressed concern that AI may replace certain healthcare jobs, indicating a need for ongoing dialogue about the implications of AI in the workforce. This mixed outlook reflects the interns' understanding of AI's potential benefits and challenges as they prepare to enter a rapidly evolving healthcare landscape.

**Table 5:** Future perspectives on AI in healthcare (N=349) Data are represented as n (%), where n indicates the number of participants and % represents the percentage of the total sample (N=349) AI: artificial intelligence

Perspective statement	Strongly disagree (n, %)	Disagree (n, %)	Neutral (n, %)	Agree (n, %)	Strongly agree (n, %)
AI will significantly change healthcare roles	25 (7.2%)	45 (12.9%)	60 (17.2%)	90 (25.8%)	129 (36.9%)
AI will replace certain healthcare jobs	50 (14.3%)	70 (20.1%)	40 (11.5%)	90 (25.8%)	99 (28.4%)

## Discussion

This study aimed to explore the perceptions of newly graduated interns in Bahrain regarding the use of artificial intelligence (AI) in medicine. The findings reveal a nuanced understanding of AI among the participants, highlighting both optimism for its potential benefits and concerns about its implications for the healthcare workforce.

The results indicate varying levels of awareness and familiarity with AI among the interns. While a majority (146, 41.8%) identified as being familiar with AI, a significant portion (65, 18.6%) reported being not familiar at all. This disparity suggests that while many interns recognize the importance of AI, there is a critical need for enhanced educational initiatives in medical training to bridge this knowledge gap [8-11]. The integration of AI-focused curricula in medical schools could better prepare future healthcare professionals to navigate and utilize these technologies effectively [5,12].

A substantial number of participants expressed positive perceptions regarding the impact of AI on patient care. The belief that AI improves diagnosis (114, 32.7%) and assists in reducing medical errors (129, 36.9%) reflects a growing confidence in AI's capabilities to enhance clinical outcomes. These findings are consistent with existing literature that highlights AI's potential to augment diagnostic accuracy and improve patient management [10-14]. However, it is crucial to consider that while AI presents significant opportunities, its implementation must be approached cautiously, with a focus on maintaining the human element of care and addressing potential biases in AI algorithms [3,11].

Despite the optimism, the study uncovered notable concerns regarding AI's role in healthcare. The predominant worries are related to data privacy and security (140, 40.1%) and job displacement (110, 31.5%). These concerns are valid, as the integration of AI systems can lead to ethical dilemmas and fears about job security among healthcare professionals. It is essential for policymakers and healthcare institutions to address these concerns proactively by establishing robust data protection measures and ensuring that AI serves as a tool to support, rather than replace, healthcare workers [5,9]. Engaging healthcare professionals in discussions about the ethical implications of AI can foster a collaborative environment that prioritizes patient welfare [12-15].

The findings indicate a significant gap in training related to AI technologies, with 189 participants (54.2%) reporting no training received. This lack of preparedness raises important questions about how newly graduated interns can effectively engage with AI in their practice [2-8]. As AI continues to evolve, it is imperative that medical training programs incorporate comprehensive training on AI applications, equipping future physicians with the skills necessary to harness these technologies in clinical settings. Furthermore, ongoing professional development opportunities should be made available to ensure that current practitioners remain informed about advancements in AI [3,9].

The majority of participants (129, 36.9%) strongly believed that AI will significantly change healthcare roles, reflecting an awareness of the transformative potential of technology in the medical field. However, the concern that AI may replace certain jobs (99, 28.4%) underscores the need for a balanced approach to integrating AI into healthcare. By emphasizing the collaborative potential of AI and highlighting the irreplaceable qualities of human caregivers, stakeholders can work to alleviate fears while promoting a vision of AI as a partner in healthcare delivery [11-14].

Limitations

While this study provides valuable insights, it is essential to acknowledge its limitations. The reliance on self-reported data may introduce bias, and the findings may not be generalizable beyond the specific context of Bahrain. Future research should consider longitudinal studies that track changes in perceptions over time, as well as qualitative approaches to gain deeper insights into the experiences and attitudes of healthcare professionals regarding AI.

## Conclusions

In conclusion, this study highlights the diverse perceptions of newly graduated interns in Bahrain regarding artificial intelligence in medicine, revealing a promising awareness of its potential benefits alongside significant concerns about data privacy and job displacement. The findings underscore the urgent need for enhanced training and education in AI technologies within medical curricula, ensuring that future healthcare professionals are adequately prepared to navigate the evolving landscape of healthcare. By addressing both the opportunities and challenges presented by AI, stakeholders can foster a collaborative environment that enhances patient care while maintaining the essential human element in medical practice.

## References

[1] Mir MM, Mir GM, Raina NT (2023). Application of artificial intelligence in medical education: current scenario and future perspectives. J Adv Med Educ Prof.

[2] Chen M, Zhang B, Cai Z (2022). Acceptance of clinical artificial intelligence among physicians and medical students: a systematic review with cross-sectional survey. Front Med (Lausanne).

[3] Guerra GA, Hofmann H, Sobhani S (2023). GPT-4 artificial intelligence model outperforms ChatGPT, medical students, and neurosurgery residents on neurosurgery written board-like questions. World Neurosurg.

[4] Tolentino R, Baradaran A, Gore G, Pluye P, Abbasgholizadeh-Rahimi S (2023). Curriculum frameworks and educational programs in artificial intelligence for medical students, residents, and practicing physicians: a scoping review protocol. JBI Evid Synth.

[5] Tolentino R, Baradaran A, Gore G, Pluye P, Abbasgholizadeh-Rahimi S (2024). Curriculum frameworks and educational programs in AI for medical students, residents, and practicing physicians: scoping review. JMIR Med Educ.

[6] Narayanan S, Ramakrishnan R, Durairaj E, Das A (2023). Artificial intelligence revolutionizing the field of medical education. Cureus.

[7] Bohler F, Aggarwal N, Peters G, Taranikanti V (2024). Future implications of artificial intelligence in medical education. Cureus.

[8] Krive J, Isola M, Chang L, Patel T, Anderson M, Sreedhar R (2023). Grounded in reality: artificial intelligence in medical education. JAMIA Open.

[9] Chen JX, Bowe S, Deng F (2024). Residency applications in the era of generative artificial intelligence. J Grad Med Educ.

[10] Jackson P, Ponath Sukumaran G, Babu C (2024). Artificial intelligence in medical education - perception among medical students. BMC Med Educ.

[11] Hu R, Fan KY, Pandey P (2022). Insights from teaching artificial intelligence to medical students in Canada. Commun Med (Lond).

[12] Daher OA, Dabbousi AA, Chamroukh R, Saab AY, Al Ayoubi AR, Salameh P (2024). Artificial intelligence: knowledge and attitude among Lebanese medical students. Cureus.

[13] Umer M, Naveed A, Maryam Q, Malik AR, Bashir N, Kandel K (2024). Investigating awareness of artificial intelligence in healthcare among medical students and professionals in Pakistan: a cross-sectional study. Ann Med Surg (Lond).

[14] AlZaabi A, AlMaskari S, AalAbdulsalam A (2023). Are physicians and medical students ready for artificial intelligence applications in healthcare?. Digit Health.

[15] Liu DS, Sawyer J, Luna A (2022). Perceptions of US medical students on artificial intelligence in medicine: mixed methods survey study. JMIR Med Educ.

